# Multiomics analysis reveals microbial diversity and activity through spontaneous fermentation of *Theobroma cacao*

**DOI:** 10.1016/j.heliyon.2024.e40542

**Published:** 2024-11-19

**Authors:** Ynara da Costa Fonseca, Celina Eugenio Bahule, Hector Herrera, Luiza Helena da Silva Martins, Alessandra Santos Lopes, Juliana Silva Cassoli, Felipe Costa Trindade, Isa Rebecca Chagas da Costa, Paulo Henrique de Oliveira Costa, Guilherme Oliveira, Rafael Borges da Silva Valadares

**Affiliations:** a Graduate Program in Agricultural Applied Biotechnology, Federal Rural University of Amazonia, President Tancredo Neves Ave, 2501, Belém, CEP 66.077-830, Brazil; b Vale Institute of Technology, Boaventura da Silva Street 955, Belém, CEP 66050-090, Brazil; c Center of Studies in Science and Technology (NECET), Rovuma University, Niassa Branch, Lichinga, Mozambique; d Departamento de Ciencias Forestales, Facultad de Ciencias Agropecuarias y Medioambiente, Universidad de La Frontera, Temuco, 4811230, Chile; e Center for Biodiversity and Ecological Sustainability (C-BEST), Facultad de Ciencias Agropecuarias y Medioambiente, Universidad de La Frontera, Chile; f Institute of Animal Health and Production, Federal Rural University of Amazonia, President Tancredo Neves Ave., 2501, Belém, CEP 66077-830, Brazil; g Faculty of Food Engineering, Institute of Technology, Federal University of Pará, Belém, CEP 66075-110, Brazil; h Laboratory of Omics Sciences, Institute of Biological Sciences, Guamá Campus, Federal University of Pará, 66075-110, Belém, Brazil

**Keywords:** Bacteria, Cocoa, Metabarcoding, Metaproteomics

## Abstract

To gain insight into the active microbiota during spontaneous fermentation of *Theobroma cacao* L., this study assessed protein diversity during 120 h using a combined metabarconding and metaproteomics approach. During the first days of fermentation, most of the peptides were associated with *T. cacao* and yeast (0–72 h). Peptides associated with bacteria became more abundant after 72 h of fermentation, coinciding with a decrease in peptides associated with cocoa (96–120 h). In addition to the known microorganisms involved in fermentation, such as *Saccharomyces*, *Lactobacillus* and *Acetobacter*, novel genera were also metabolically active, including *Microvirga*, *Inquilinus*, *Candolleomyces* and *Lasiodiplodia*.. The results showed a consistency in the main genera detected by both techniques, but the identification of unexplored genera such as *Inquilinus*, *Microvirga*, *Cyphellophora* and *Ashbya gossypii*, among others, suggests that this omics approach needs to be used together for more comprehensive results on spontaneous fermentation. In conclusion, studies combining techniques such as metabarcoding and metaproteomics should be considered in fermentation studies, as a single technique would result in omissions regarding the activity of certain microorganisms that may be important for the course of spontaneous fermentation.

## Highlights

• Microbial diversity was identified by metaproteomics and metabarcoding.
• Bacterial proteins significantly increase during the final stages of fermentation.
• Novel taxa such as *Ferruginibacter*, *Dongia*, *Inquilinus* and *Microvirga* were active during fermentation.
• Metaproteomics and metabarcoding should be used together to obtain comprehensive data on cocoa fermentation.

## 1 Introduction

*Theobroma cacao* L. (cocoa) is a native tropical fruit tree cultivated in Asia, Africa, and South America in areas known as the cocoa belt [1,2]. Brazil is the largest producer of cocoa in South America, with an estimated 239,400 dry tons, accounting for nearly 5 % of global cocoa production [[3], [4], [5]]. In northern Brazil, the state of Pará has become one of the largest producers, with a production of 145,000 tons of cocoa beans per hectare, making cocoa the second largest commodity in the Pará economy [4]. An agroforestry system developed by local farmers, where cocoa is grown alongside other crops, can directly influence the organoleptic characteristics of cocoa [4]. In addition, in many places, cocoa production by indigenous populations is a hands-on process. Therefore, understanding how local microbiota can contribute to improving the quality of cocoa-derived products is fundamental.

Culture-dependent methods provide a wide range of information on the microbial diversity associated with cocoa fermentation [[6], [7], [8]]. The main microbes involved in spontaneous fermentation include yeasts, lactic acid bacteria, and acetic acid bacteria [9]. Evidence suggests that yeasts are dominant in the anaerobic conditions of the first 24 h [9,10]. They mainly metabolize glucose, fructose and citric acid to produce ethanol and carbon dioxide [11,12]. The pectin in the pulp is hydrolyzed, allowing for aeration and further activity of lactic and acetic acid bacteria [13,14]. The ethanol is metabolized under aerobic conditions, forming acetic acid, destabilizing the cotyledons, and initiating protein, polyphenol, and lipid modifications that produce flavor and aroma precursors [15]. These modifications induce the interaction of amino acids with polyphenols, affecting the color of the final product [15]. At the same time, seed-derived metabolites and the activity of enzymes such as polyphenol oxidases, invertases, and proteases are directly related to the characteristic flavor and aroma of fermented cocoa [16,17]. Many of these studies have been conducted using conventional methods to analyze microbial diversity in spontaneous fermentation, typically focusing on the selection of specific target microorganisms. However, such techniques may exclude non-viable, low abundance, non-culturable, or metabolically codependent microorganisms [18,19].

As metabarcoding approaches, culture-free methods explain community changes based on the relative abundance of specific DNA markers, but do not distinguish between active and inactive structures [20,21]. Proteomic-based technologies are an essential complementary resource that have become valuable tools for the analysis of spontaneous fermentation, where the focus is on functional screening of active microorganisms in fermented products [18]. Therefore, gaining more information about the diversity of active microorganisms and the proteins they express can shed light on critical aspects of cocoa bean processing. In addition, peptidome analysis can reveal taxonomical identity coupled with functional analysis, directly linking microbial taxa to processes. With this strategy, we can clearly point to key microorganisms that could be managed to enhance a selected trait.

A new approach to the study cocoa will combine proteomics and metabarcoding, which has the advantage of allowing the study of fermentative microorganisms. The design of this study was based on the assumption that there is complementarity between these two omics techniques and that this will ensure consistency of results, increase accuracy and reduce time. Therefore, this study aimed to assess the diversity and activity of microorganisms in spontaneous fermentation using a combined metabarcoding and metaproteomic approach. Identification with these combined methods will open doors for a better understanding of the establishment of links between microbial metabolism and the final quality and sensory characteristics of cocoa-derived products.

## 2 Materials and methods

### 2.1 Collection of cocoa beans

The Forastero *cocoa* beans were fermented in wooden boxes and dried on a farm in Tomé-Açu, PA, Brazil (2°28′41.3″S 48°16′50.7″W) in May 2019. The temperature varies between 32 °C (day) and 23 °C (night). The process was carried out as described by Konagano et al. [22] using 2 troughs containing approximately 60 kg of seeds and covered with banana leaves. The fermentation lasted 120 h (five consecutive days) and the temperature was monitored throughout the process. The almonds were then dried for 96 h in the producer's natural drying oven. The troughs went through all the stages of the fermentation process, such as aeration or homogenization of the almonds every 24 h until the end of the process. Composite samples of about 100 g of samples were taken from these troughs at 0, 24, 48, 72, 96 and 120 h. The samples were stored in sterile polyethylene packaging at refrigeration temperatures (4–8 °C) and sent to the Laboratory of Biotechnological Processes, where the physicochemical analyses were carried out. Similarly, proteomic determinations were performed in the Laboratory of Omics Sciences, Institute of Biological Sciences, Federal University of Pará.

### 2.2 Physico-chemical analyses

The physicochemical analyses of the fermented cocoa beans from 0 h to 120 h were carried out according to the protocol of Horwitz [23]. For this purpose, the cocoa beans were previously crushed in a manual mill. The analyses of moisture (method 963.15), pH (method 970.21), total titratable acidity (TTA, method 31.06.06), and total reducing sugars (TRS) were performed according to the dinitrosalicylic acid (DNS) method described by Miller [24]. All analyses were performed in triplicate.

### 2.3 Fungal and bacterial metabarcoding

#### 2.3.1 DNA extraction

Total DNA was isolated from 0.25 mg of macerated *cocoa* beans collected at different stages of fermentation using the PowerSoil® DNA Isolation Kit (QIAGEN, Hilden, Germany) according to the manufacturer's recommendations. Total DNA concentration was estimated using the Qubit™ dsDNA HS Assay (Thermo Fisher Scientific, Waltham, MA, USA), and DNA quality was checked in 1 % agarose gel electrophoresis.

#### 2.3.2 16S rRNA and 18S rRNA gene sequencing

The 16S rRNA gene was amplified by PCR using the bacterial primer set S-D-Bact-0341-b-S-17-N (5″-TCGTCGGCAGCGTCAGATGTGTATAAGAGACAGCCTACGGGNGGCWGCAG-3″) and S-D-Bact-0785-a-A-21-N (5″-GTCTCGTGGGCTCGGAGATGTGTATAAGAGACAGGACTACHVGGGTATCTAATCC-3″), according to the PCR conditions described in Costa et al. [25]. Similarly, the ITS region of the 18S rRNA gene was amplified by PCR using the primer set fITS7i (5″-TCGTCGGCAGCGTCAGATGTGTATAAGAGACAGGTGARTCATCGAATCTTTG-3″) and ITS4i (5″-GTCTCGTGGGCTCGGAGATGTGTATAAGAGACAGTCCTCCGCTTATTGATATGC-30), following the PCR conditions described in Costa, Nascimento, Herrera, Gastauer, Ramos, Caldeira, Oliveira and Valadares [25]. Amplicon size and quality were assessed on an Agilent TapeStation (Agilent Technologies, Santa Clara, CA, USA) using a D1000 ScreenTape system. Libraries were purified using the AMPure XP Purification Kit (Beckman Coulter, Brea, CA, USA) and processed using the Nextera XT Kit (Illumina, San Diego, CA, USA). Gene libraries were sequenced on a Miseq-Illumina platform using a MiSeq V3 reagent kit (600 cycles; Illumina). Raw data from this study were submitted to the NCBI Sequence Read Archive (http://trace.ncbi.nlm.nih.gov/Traces/sra/) under accession number PRJNA816498 (https://www.ncbi.nlm.nih.gov/bioproject/PRJNA816498/).

The resulting ITS and 16S sequences were analyzed using the Pipeline for MetaBarcoding Analysis (PIMBA) [26,27]. Briefly, low quality sequences were filtered and trimmed using PRINSEQ v0.20.4, and forward and reverse sequences were merged using PEAR v0.9.19 [28]. Reads were dereplicated, singletons were removed, and sequences were truncated to 200 for fungi and 240 for bacteria. Chimeras were filtered and sequences were grouped into operational taxonomic units (OTUs) using VSEARCH v2.8.2. Taxonomic assignments were made using the UNITE database for fungi and the Ribosomal Database Project for bacteria [29,30].

Graphs were constructed considering alpha and beta diversity in R software using the ggplot2 and vegan packages. Beta diversity was calculated, and principal coordinate analysis (PCoA) graphs were constructed using the “weighted UniFrac distances” in R software using the phyloseq package. Heatmaps were constructed with the total abundance of OTUs using R software (packages pheatmap and phyloseq). Alpha diversity was estimated using the vegan package with Shannon and Simpson diversity indices.

### 2.4 Metaproteomic profile

#### 2.4.1 Protein extraction

Protein isolation was carried out according to Wang et al. [31]. A total of 3 g of cocoa seeds collected at different stages of fermentation were macerated in liquid nitrogen. Then 9.0 mL extraction buffer [0.85 M sucrose, 0.1 M Tris-HCl (pH 8.0), 2 % (w/v) sodium dodecyl sulfate (SDS), 1 mM phenylmethylsulfonyl fluoride and 2 % (w/v) polyvinylpolypyrrolidone] was added to the macerated seeds. Subsequently, 3 mM Protease Inhibitor Cocktail Powder (Sigma-Aldrich, St. Louis, MO, USA) and 70 mM dithiothreitol (DTT) were added. Samples were incubated for 10 min at room temperature and then sonicated five times (30 s duration for each event and 30 s intervals). Each sample was fractionated into eight microtubes and homogenized individually with 700 μl of saturated phenol (pH 8.0).

After centrifugation at 14,000 rpm for 7 min at 4 °C, the phenolic phase of each aliquot was collected and combined in new microtubes, followed by further centrifugation at 14,000 rpm for 7 min at 4 °C to remove any residual SDS or aqueous phase. The phenolic phase was collected and proteins were precipitated by incubating the samples overnight at −80 °C with 800 μl of 0.1 M ammonium acetate (prepared in absolute methanol at −20 °C).

The samples were centrifuged at 14,000 rpm for 4 min at 4 °C and the supernatant was discarded. The remaining protein pellet was washed with 1.5 mL of 80 % (v/v) ice-cold acetone and ethanol. Finally, the resulting pellet was dried in a vacuum concentrator for 7 min. Protein extracts were solubilized by the addition of 100 μl of 0.1 % RapiGest™ Surfactant (Waters, Milford, MA, USA) and stored at −80 °C until protein digestion. Protein concentration was estimated using a Qubit 3.0 Fluorometer (Invitrogen, Waltham, MA, USA).

#### 2.4.2 Protein digestion and sample desalting

Approximately 50 μg of proteins were quantified per sample. Preparation for digestion included protein reduction with DTT 5 mM and incubation for 25 min at 56 °C, followed by alkylation with iodoacetamide (IAA) 14 mM for 30 min at room temperature. Residual IAA was removed by the addition of DTT (5 mM) and incubation for 15 min at room temperature, followed by the addition of calcium chloride (1 mM) and treatment with trypsin (20 ng μl^−1^) for 20 h at 37 °C. Trifluoroacetic acid was then added to a final concentration of 0.4 % to stop the enzymatic reaction. The samples were incubated at 37 °C for 90 min and then centrifuged at 14,000 rpm at 4 °C for 10 min. The supernatant was then transferred to appropriate vials. The pH of the solution was adjusted to 10 with 1 N ammonium hydroxide for effective separation on the first dimension column of ultra-performance liquid chromatography (UPLC).

#### 2.4.3 Protein identification and bioinformatics analysis

Protein identification and quantification were performed according to the methodology described in Nascimento et al. [27], with minor modifications. Samples were analyzed in a microUPLC tandem nanoESI-Q-TOF platform using a 1D-RP Acquity UPLC M-Class system coupled to a Xevo-G2 XS mass spectrometer (Waters Corporation, Milford, MA, United States). Peptide samples (200 ng) were loaded onto an M-Class HSS T3 column (100 Å, 1.8 mm, 75 mm × 150 mm, Waters Corporation, Milford, MA, United States). Fractionation was performed using an acetonitrile gradient of 3–40 % (v/v) over 120 min at a flow rate of 300 nL/min directly into the Xevo-G2 XS mass spectrometer. For each measurement, MS and MS/MS data were acquired in positive resolution mode using the MSE approach with a resolution of approximately 40,000 FWHM and over a 50–1990 *m*/*z* range. The spectral acquisition time in each mode was 0.5 s with an interscan delay of 0.01 s, resulting in a total cycle time of 1.1 s to acquire one cycle of low and high energy data. The lock mass channel was sampled every 30 s. The mass spectrometer was calibrated using a phosphoric acid (686.8461 *m*/*z*) solution delivered through the reference nebulizer of the NanoLock spray source. Raw data were processed using Progenesis QI software v.2.0 (Waters), as reported in Nascimento et al. [27].

A FASTA database was constructed by retrieving bacterial and fungal protein sequences from NCBI based on the taxonomic list generated by 16S and ITS sequencing. Sequences from *Theobroma cacao* (also retrieved from NCBI) were then added to the same FASTA file. Searches were performed in Progenesis QIP (Nonlinear Dynamics) and proteins were accepted with at least 96 % probability. Functional and taxonomic analysis of the assigned proteins was performed using Unipept v.4.0 software. Differences between samples were estimated on the basis of functional or taxonomic composition using the Unipept desktop application [32].

### 2.5 Statistical analyses

The results of physicochemical analyses were subjected to analysis of variance (ANOVA), and means were compared using Tukey's test (p < 0.05). Principal component analysis (PCA) of the metaproteome was performed using R software (R Core Team 2018; https://www.R-project.org). Differences between total sequence variables and total identified peptides were detected by one-way ANOVA. Post hoc pairwise comparisons were performed using Tukey's multiple range test. All statistical tests were performed using R software (R Core Team 2018; https://www.R-project.org), with statistical significance set at p < 0.05.

## 3 Results and discussion

This study characterizes the microbial diversity associated with the spontaneous fermentation of cocoa beans in the eastern Amazon, identifying specific changes in the abundance of microorganisms and the variety of proteins they express. Metaproteomics proves to be a comprehensive approach, revealing not only the diversity but also the activity of microorganisms during the spontaneous fermentation of cocoa beans. The results are similar to those obtained by metabarcoding but additionally reveal active taxa with a limited reported role in cocoa fermentation.

### 3.1 Physico-chemical analyses

The pH was variable during fermentation, with higher values in samples between 72 h and 96 h, with a tendency similar to that found in TTA (Table 1). The moisture of the samples varied from 37.46 at 24 h to 42.86 at 120 h (Table 1). The TRS mainly showed a constant decrease, with values of 0.49 after 120 h of fermentation (Table 1).

**Table 1:** Physico-chemical characteristics of fermented cocoa beans from Tomé-Açu evaluated at different days of fermentation.

Fermentation time	pH	TTA (meq·kg^−1^)	Moisture (%)	TRS (mg/g)
0 h	5,63 ± 0,02^d^	7,07 ± 0,64^c^	39,66 ± 0,23^e^	1,51 ± 0,02^a^
24 h	5,83 ± 0,01^c^	5,93 ± 0,00^e^	37,46 ± 2,05^f^	1,40 ± 0,005^b^
48 h	4,02 ± 0,01^f^	3,93 ± 0,00^f^	41,80 ± 0,59^c^	1,00 ± 0,004^c^
72 h	10,00 ± 0,005^a^	20,12 ± 0,37^a^	41,69 ± 0,15^d^	0,80 ± 0,03^e^
96 h	7,06 ± 0,02^b^	15,04 ± 0,11^b^	42,69 ± 0,40^b^	0,88 ± 0,06^d^
120 h	5,08 ± 0,01^e^	6,00 ± 0,00^d^	42,86 ± 0,19^a^	0,49 ± 0,006^f^

∗Values represent mean ± standard deviation. Different letters in superscript in the same line indicate statistically different values (p ≤ 0.05). Total titratable acidity is expressed as milliequivalents of NaOH.

The results show a tendency towards anaerobic and aerobic fermentation. Ethanol production by yeasts and fungi occurs within the first 48 h and is then metabolized to acetic acid by acetic acid bacteria, reaching its peak at 72 h [8,33]. At the same time, lactic acid, which is produced by lactic acid bacteria (LAB) from 24 h onwards, along with other acids such as citric acid and malic acid, also reaches its maximum level at 72 h. This process contributes to the high TTA value observed (Table 1). The decrease in acid concentration after 96 h is mainly due to evaporation with increasing temperature and also to aeration of the fermentation mass [8].

The pH values found were not statistically different from each other (p ≥ 0.05), the results found are similar to other studies [22], and the values found (4–5) are relevant because they allow the activity of proteases that are important in the formation of chocolate aroma [34]. However, the pH value observed after 72 h (Table 1) showed a significant increase. This phenomenon can be explained by the formation of weak acid salts (acetic and lactic), which cause pH variations due to the partial neutralization of H⁺ ions [35].

Regarding reducing sugars, the values found were not statistically different (p ≥ 0.05), and there was a significant decrease during the process, with a small increase in the 96-h period (Table 1), this fact may be related to the hydrolysis of sucrose into glucose and fructose by lactic and acetic acid present in the medium, and these monosaccharides are available for the fermentation process [36].

The results obtained for the moisture analysis (Table 1), are similar to the standards established by Ferreira [37], in which during the fermentation process the mass of almonds has 40–50 % water, and at the end of the process it is necessary to dry this mass to reduce the water concentration to 7–8 % (important to prevent insect attack and proliferation of fungi), and the results were not statistically different (p ≥ 0.05).

### 3.2 Fungal and bacterial diversity

**Fig. 1:**
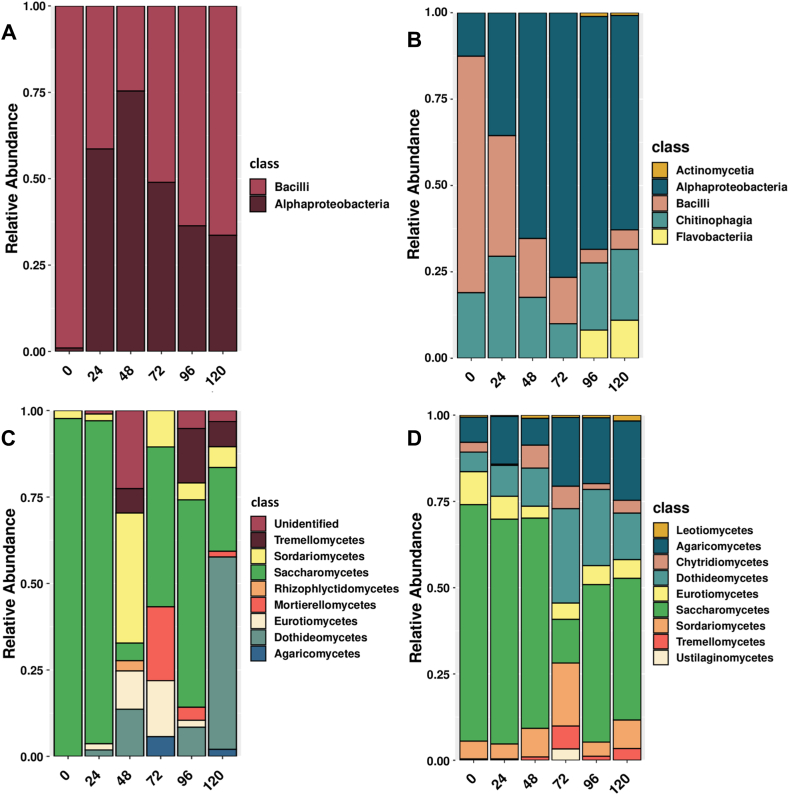
Main classes of bacteria (A and B) and fungi (C and D) identified by metabarcoding (left) and metaproteomics (right).

**Fig. 2:**
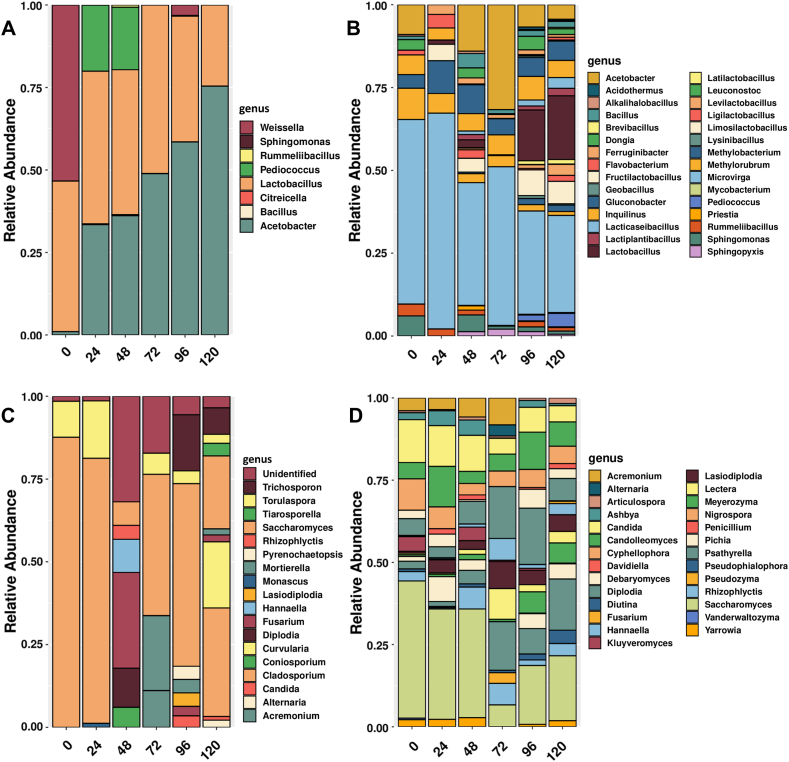
Main genera of bacteria (A and B) and fungi (C and D) identified by metabarcoding (left) and metaproteomics (right).

In this study, two omics techniques were combined for the purpose of complementarity in the interpretation of cocoa fermentation events during 120 h. It was found that the two techniques agreed in identifying the main classes and genera of fungi and bacteria active during fermentation, supporting the applicability of both techniques for studying the microbial profile in fermented cocoa (Fig. 1, Fig. 2). However, there are also clear differences in their performance, as metaproteomics can also provide information on proteins expressed by microorganisms active during fermentation. This study is one of the few that had used different omics analysis techniques to understand the microbial diversity in a fermented product.

Our results showed that metabarcoding identified two classes of bacteria (Alphaproteobacteria and Bacilli), but using metaproteomics it was possible to identify other classes not detected by metabarcoding, namely Actinomycetia, Chitinophagia and Flavobacteriia (Fig. 1A and B). The relative abundance of fungal proteins showed that the classes Saccharomycetes, Eurotiomycetes, Dothideomycetes and Agaricomycetes were the most active and were also identified as the most abundant in metabarcoding (Fig. 1C and D). While metaproteomics provides a broad spectrum in the identification of microorganisms involved in fermentation, metabarcoding still leaves a good part of the microbiota unidentified. These differences can be due to some factors: i) DNA recovery is a sensitive technique and there can be a significant loss of information, from the treatment of the sample to the connection with the database; ii) it is likely that the presentation of the sample (solid) for analysis or the stages of treatment of this sample, have influenced the loss of some genetic material or the failure to read it during the sequencing process; iii) DNA metabarcoding is highly biased by PCR efficiency, while metaproteomics relies on the ability to recover peptides in high abundance (without any sort of signal amplification), so it is likely that overall response of both techniques should differ.

In terms of genera, our results showed that metaproteomics was able to identify about 30 genera of bacteria, while only 8 genera were identified by metabarcoding (Fig. 2A and B). This discrepancy between the two methods can also be seen in fungal genera, where 27 genera were reported by metaproteomics compared to 17 by metabarcoding (Fig. 2C and D). It should be noted that the common and most abundant genera such as *Lactobacillus*, *Acetobacter* and *Saccharomyces* were easily detected by both techniques.

**Fig. 3:**
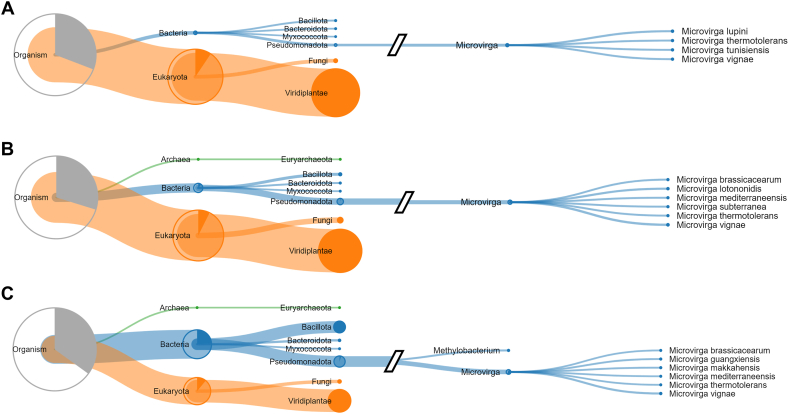
Peptide-based dendrogram built by Unipept 4.0 using all peptides obtained from cocoa fermentation with significant protein matches at 24 h (A), 72 h (B) and 120 h (C). The phylogenetic tree was constructed using the least common ancestor (LCA) method. The sizes of the circles refer to the abundance of peptides for each taxonomic level.

Only 6 bacterial genera coincided between the techniques (*Bacillus*, *Lactobacillus*, *Pediococcus*, *Sphingomonas*, *Rummelibacillus* and *Acetobacter*), of which there was no clear trend of occurrence according to fermentation time (Fig. 2A and B). Looking at the most abundant bacterial genera, *Microvirga* and *Acetobacter* were present throughout the fermentation process and were detected by metaproteomics. Particularly important is the case of *Microvirga*, which was active throughout the fermentation (Fig. 3). Similarly, *Pediococcus* was detected by metabarcoding after 96 h and this was the case for both techniques. The genus *Weisella*, which was detected by metabarcoding before fermentation and also in the first 24 h, was not found by proteomic analysis, probably due to lower metabolic activity and protein accumulation of this genus. It is clear to see that most of the genera detected by the metabarcoding technique are exactly those that appear in considerable relative abundance in the metaproteomic analysis. Some studies suggest that using metabarcoding data can generate a functional screen showing the activity of microorganisms during fermentation [18]. Our study proves that there may be a margin of error when considering such a feature, as it was shown that metabarcoding does not detect individuals with low abundance in the sample, even if they played a role in the process. Likewise, abundant individuals without a direct role can be easily detected by this technique. The genus *Rummelibacillus*, which was present throughout fermentation according to metaproteomics, was only detected by metabarcoding after 72 h of fermentation, probably because its abundance increased over time until it reached a sufficient level for detection. In contrast, *Methylorubrum* and *Methylobacterium* were detected by metaproteomics, again demonstrating the value of the technique in relation to the amount of material. *Methylorubrum* and *Methylobacterium* have been detected in fermented products and may play a role in the production of odorants [38]. Therefore, it is important to screen and identify the beneficial taxa that help improve the sensory characteristics of fermented products.

The same tendency is seen in the analysis of the fungal genera, where it is possible to see that *Saccharomyces*, *Fusarium*, *Candida*, *Torulospora*, among others, are commonly found. Regarding the fungal genera detected by metabarcoding and therefore present in the metaproteomics, we can assume that contamination probably occurred during fermentation and the source could be utensils, handlers or even the environment itself. Also, considering that fungi are versatile and can easily contaminate the environment. Species of the genus *Saccharomyces* are recognized as important contributors to cocoa bean fermentation, especially in the production of aromatic compounds [8,39,40]. Moreover, they play a crucial role in the degradation of fermentable sugars present in the mucilaginous pulp during the initial stages of fermentation [40,41]. In addition, *Torulaspora* showed a significant abundance at 0 h and 24 h (Fig. 2C). *Torulaspora* has also been recognized as an important participant in cocoa bean processing, potentially influencing the sensory properties of fermented products [42]. The significant presence of yeast genera in our study may have a positive impact on the fermentation process, as co-inoculation of *Saccharomyces* and *Torulaspora* has previously been shown to improve the chemical properties of the resulting products [43]. Similarly, the yeast *Trichosporon*, while known for its importance in oilseed fermentation [44], requires further investigation to determine its direct effects on the spontaneous fermentation of cocoa beans as no proteins were associated to this taxa (Fig. 2C and D).

### 3.3 Metaproteomics as a tool to unravel microbial diversity and relative activity in food biochemistry

In this study, metaproteomics and metabarcoding were considered as complementary techniques, where one allows the complete analysis of the players in fermentation through DNA analysis, while in the other, the identified peptides provide the functional profile of the identified microorganisms. A total of 36801peptides assigned to 807 proteins were identified in cocoa fermentation (Dataset S1). Of these proteins, 250 were assigned to *T. cacao*, 70 to yeasts, 97 to filamentous fungi and 390 to bacteria (Dataset S1). These proteins were associated with 57 different genera, 30 bacteria, 12 yeasts and 15 filamentous fungi (Dataset S1).

**Fig. 4:**
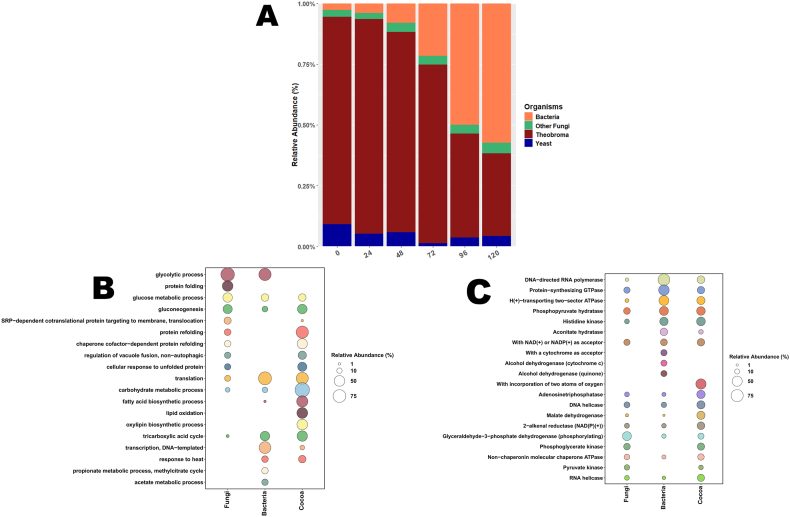
(A) Relative abundance of proteins associated with cocoa beans and their microbial community at different times during the spontaneous fermentation process. (B) Functional categorization of peptides in terms of Gene Ontology biological process and (C) EC number through the spontaneous fermentation of cocoa beans.

The dynamics of total peptides during spontaneous fermentation was variable. In the early stages, peptides were mainly associated with *T. cacao*, yeasts and filamentous fungi, while in the later stages they were mainly associated with bacteria (Fig. 4). The decrease in the relative abundance of *T. cacao* peptides is related to their degradation by fermentation. The highest number of peptides attributed to bacteria was detected especially at 48 and 72 h, when their relative abundance increased (Fig. 4). In contrast, the peptides attributed to fungal taxa were lower in the final stages of fermentation (Fig. 4).

In spontaneous fermentation, the microorganisms that participate in fermentation are expected to come from a variety of uncontrolled sources, from the environment in which the raw material was harvested, from the conditions of transportation, storage, handling, the fermentation site, the utensils used. and even the operators. This makes it difficult to predict the microbiota that will participate in fermentation, as well as the functionality of each species present [19]. While metabarcoding revealed significant fluctuations in the abundance of microorganisms, at different time points during spontaneous fermentation, it is important to note that this abundance does not necessarily correlate with metabolically active microorganisms (Dataset S1). The metaproteomic approach revealed that many of the expressed proteins were primarily associated with the internal metabolism of microorganisms and did not directly contribute to the fermentation process (Dataset S1). However, our analysis identified enzymes critical for fermentation, including glyceraldehyde-3-phosphate dehydrogenase, enolase, triosephosphate isomerase, and phosphoglycerate kinase [45,46]. Among the identified microorganisms, we highligh the role of the yeasts *S. cerevisiae*, and *K. marxianus* throughout spontaneous fermentation (Dataset S1), but also reveal the activity of novel microbial taxa with putative beneficial roles in spontaneous fermentation, including bacteria (e.g., *Ferruginibacter* sp., *Dongia mobilis*, *Inquilinus* spp., *Microvirga* spp., *Rummeliibacillus* spp. and *Sphingopyxis* spp.), yeast-like fungi (e.g., *Cyphellophora europaea*), and filamentous fungi (e.g., *Acremonium chrysogenum*, *Ashbya gossypii*, *Candolleomyces aberdarensi*, *Lasiodiplodia theobromae*, and *Psathyrella* spp.). This implies that multiple microorganisms are involved in fermentation, as these enzymes are not exclusive to a particular group of microorganisms. Glycolysis, a central component of the alcoholic fermentation pathway, relies on these enzymes, which are produced as a result of the metabolic activity of microorganisms [47]. In this study, several microorganisms were found to express these key enzymes (i.e. glyceraldehyde-3-phosphate dehydrogenase, enolase, triosephosphate isomerase, and phosphoglycerate kinase) throughout the fermentation process, but they were associated with different taxa (Dataset S1).

The majority of the results are consistent with existing research and confirm the involvement of specific microorganisms such as *S. cerevisiae*, *Acetobacter* spp. and *Lactobacillus* spp. in cocoa fermentation. However, our results also shed light on several strains that, although used as starters and detected by metabarcoding, did not show active proteins related to either fermentation or internal metabolism according to the particular fermentation conditions of this study (e.g. *Torulaspora*). Interestingly, certain strains, such as *Levilactobacillus* spp. *Gluconobacter* spp. *Methylorubrum* spp. and *Diutina* spp. are known for their essential roles in fermentation or are used as probiotics, but are relatively underrepresented in the literature about cocoa fermentation [38,48]. In addition, our study identified several previously unreported taxa associated with cocoa bean processing, including *Microvirga* spp. *A. gossypii*, *Inquilinus* spp. *Cyphellophora europaea*, *Ashbya gossypii*, *Candolleomyces aberdarensi*, and *Lasiodiplodia theobromae*. These findings underline the need for further research to isolate these strains and perform specific inoculations with these microorganisms.

Recent studies have highlighted the essential role of applying omics techniques, such as metabolomics and proteomics, to the study of fermented foods to understand how the active microbiome can influence the properties of these foods [[49], [50], [51]]. Specifically for cocoa, such studies would be instrumental in defining the precise role of the active microbiome during cocoa bean processing and whether these microbial communities have a positive or negative impact on the final quality of products derived from fermented cocoa beans. This demonstrates how metaproteomics not only complements DNA-based analyses, but can also provide further evidence of unidentified microbial activity. It is crucial to note that both metaproteomics and metabarcoding are likely to have technical limitations in terms of the amount and quality of protein or DNA extracted, which can significantly affect the data produced. Therefore, all methods require caution in the interpretation of results, especially for naturally fermented foods.

## 4 Conclusions

According to this study, the complementarity between omics techniques describes the microbial communities found in fermented cocoa beans, ensures consistency and accuracy of results, and reduces the time required for analysis compared to traditional culture methods. The results of the DNA analysis showed that there was significant diversity among the microorganisms. Conversely, novel taxa identified by proteomics, including *Microvirga*, *Inquilinus*, *Candolleomyces*, and *Lasiodiplodia*, were found to be active during spontaneous fermentation of cocoa beans. The combined approach of metabarcoding and metaproteomics should therefore be considered in fermentation studies, since a single technique would lead to omissions regarding the activity of certain microorganisms that may have been important for the course of spontaneous fermentation or may have influenced the observed population and, consequently, the sensory aspects of the product. New research should determine the unknown metabolic interactions between microbial communities and how they impacts cocoa quality.

## CRediT authorship contribution statement

**Ynara da Costa Fonseca:** Writing – review & editing, Writing – original draft, Investigation, Formal analysis, Data curation, Conceptualization. **Celina Eugenio Bahule:** Writing – review & editing. **Hector Herrera:** Writing – review & editing, Data curation. **Luiza Helena da Silva Martins:** Writing – review & editing. **Alessandra Santos Lopes:** Writing – review & editing. **Juliana Silva Cassoli:** Writing – review & editing, Methodology. **Felipe Costa Trindade:** Methodology, Data curation. **Isa Rebecca Chagas da Costa:** Methodology. **Paulo Henrique de Oliveira Costa:** Methodology, Data curation. **Guilherme Oliveira:** Funding acquisition. **Rafael Borges da Silva Valadares:** Methodology, Funding acquisition, Formal analysis.

## Ethical approval

This study does not contain any studies with human or animal participants performed by any authors.

## Data availability

The sequences obtained in this study were deposited in the NCBI Sequence Read Archive under the accession number PRJNA816498 (https://www.ncbi.nlm.nih.gov/bioproject/PRJNA816498/).

## Funding

This research was funded by Vale S.A., project “Cocoa project: Fermentation, pollination and bioeconomy”. G.O. is a 10.13039/501100003593CNPq fellow and received funding from 10.13039/501100002322CAPES (88887.130628/2016-00), 10.13039/501100003593CNPq (444227/2018-0, 402756/2018-5, and 307479/2016-1), and the CABANA project (10.13039/501100000690RCUK (BB/P027849/1)).

## Declaration of competing interest

The authors declare the following financial interests/personal relationships which may be considered as potential competing interests:The authors declare that they have no known competing financial interests or personal relationships that could have appeared to influence the work reported in this paper.
